# Case report: Longitudinal evaluation and treatment of a melanoma-associated retinopathy patient

**DOI:** 10.3389/fmed.2024.1445180

**Published:** 2024-09-10

**Authors:** Ryan M. Mosavi-Hecht, Paul Yang, Barrett Heyer, Christopher R. Rosenberg, Elizabeth White, Elizabeth G. Berry, Robert M. Duvoisin, Catherine W. Morgans

**Affiliations:** ^1^Department of Chemical Physiology and Biochemistry, Oregon Health & Science University, Portland, OR, United States; ^2^Casey Eye Institute, Oregon Health & Science University, Portland, OR, United States; ^3^Department of Dermatology, Oregon Health & Science University, Portland, OR, United States

**Keywords:** melanoma, MAR, autoantibodies, bipolar cells, retina, TRPM1, autoimmune retinopathy, paraneoplastic syndrome

## Abstract

Melanoma-associated retinopathy (MAR) is a paraneoplastic syndrome associated with cutaneous metastatic melanoma in which patients develop vision deficits that include reduced night vision, poor contrast sensitivity, and photopsia. MAR is caused by autoantibodies targeting TRPM1, an ion channel found in melanocytes and retinal ON-bipolar cells (ON-BCs). The visual symptoms arise when TRPM1 autoantibodies enter ON-BCs and block the function of TRPM1, thus detection of TRPM1 autoantibodies in patient serum is a key criterion in diagnosing MAR. Electroretinograms are used to measure the impact of TRPM1 autoantibodies on ON-BC function and represent another important diagnostic tool for MAR. To date, MAR case reports have included one or both diagnostic components, but only for a single time point in the course of a patient’s disease. Here, we report a case of MAR supported by longitudinal analysis of serum autoantibody detection, visual function, ocular inflammation, vascular integrity, and response to slow-release intraocular corticosteroids. Integrating these data with the patient’s oncological and ophthalmological records reveals novel insights regarding MAR pathogenesis, progression, and treatment, which may inform new research and expand our collective understanding of the disease. In brief, we find TRPM1 autoantibodies can disrupt vision even when serum levels are barely detectable by western blot and immunohistochemistry; intraocular dexamethasone treatment alleviates MAR visual symptoms despite high levels of circulating TRPM1 autoantibodies, implicating antibody access to the retina as a key factor in MAR pathogenesis. Elevated inflammatory cytokine levels in the patient’s eyes may be responsible for the observed damage to the blood-retinal barrier and subsequent entry of autoantibodies into the retina.

## 1 Introduction and case description

Melanoma-associated retinopathy (MAR) is a paraneoplastic syndrome affecting patients with cutaneous melanoma (CM) and is characterized by impairment of rod-mediated vision and the ON retinal pathway (1–4). The visual symptoms of MAR include photopsia, reduced contrast sensitivity, and progressive nyctalopia, all of which are caused by autoantibodies that bind and inhibit an essential ON-bipolar cell (ON-BC) ion channel known as TRPM1 (5, 6). This inhibition prevents ON-BC depolarization during the light response, a phenomenon that can be identified as a reduction in the b-wave amplitude of the full-field electroretinogram (ffERG) (4, 6, 7).

Melanocytes, the pigment-producing cells of the skin that become cancerous in CM, are one of few cell types outside the eye that express TRPM1 (8–11). In MAR patients, B cells produce antibodies against melanocyte antigens, including TRPM1 (5, 12, 13). Consequently, these circulating autoantibodies disrupt vision when they infiltrate the retina and bind TRPM1 in ON-BCs (6, 14). Serum screening for TRPM1 autoantibodies and ffERG testing serve as the most reliable combination of diagnostic tools for confirming MAR. However, melanoma treatment may influence the presence of serum autoantibodies or the progression of a patient’s visual symptoms over time, complicating diagnosis and prognosis (15–17). Several studies report ffERG recordings or serum autoantibody titers from a single timepoint in a patient’s disease, yet none correlate treatment data with diagnostic testing over time to assess MAR progression and resolution (16, 18–26). Here, we describe a case of bilateral MAR in a 79-year-old male with stage IV CM. In August 2021, the patient presented with unilateral vision impairment and a relative afferent pupillary defect (RAPD), prompting further ophthalmic testing and treatment for optic neuritis (Supplementary Table S1). At baseline, the patient’s intraocular pressure was 13 mmHg in both eyes. The patient was receiving pembrolizumab treatment without evidence of active disease when he developed MAR visual symptoms. After observing no response to systemic corticosteroids and severe loss of inner-retinal function on ffERG without evidence of retinal degeneration on multi-modal retinal imaging (optical coherence tomography, fundus autofluorescence, fluorescence angiography) the patient was suspected of having MAR. The diagnosis was supported by the patient’s symptoms (nyctalopia, photopsia, blue-tinted vision, and “squiggles” in his vision) and later confirmed with a positive test for anti-ON-BC autoantibodies in the serum. Unlike previous reports, we integrate the patient’s treatment history with a longitudinal evaluation of the ocular and humoral components of their disease to gain a more complete understanding of MAR pathology.

## 2 Methods

This study was performed in accordance with the Declaration of Helsinki and protection of the patient’s identity. The patient was provided with written informed consent for the use of personal medical data for scientific purposes and publication. IRB00009765. Methods details can be found in the Supplementary materials.

## 3 Diagnostic assessment and results

### 3.1 Intraocular corticosteroids restore visual function and alleviate MAR visual symptoms

**Figure 1:**
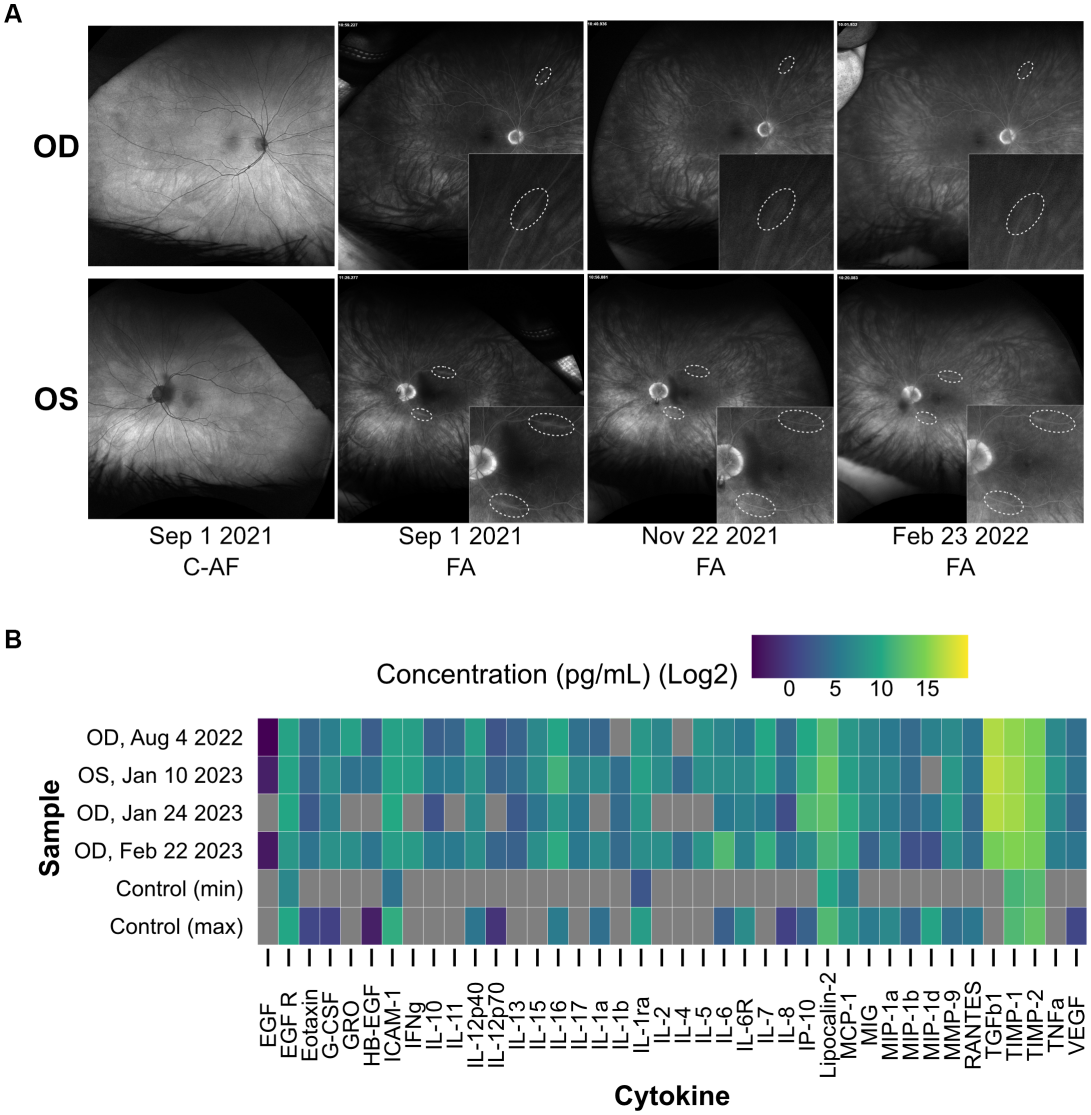
Augmented inflammatory cytokine levels and focal retinal venous leakage are detected in the MAR eye. **(A)** Baseline images of the patient’s retina (first column) show no evidence of abnormalities in color or autofluorescence (C-AF). Fluorescein angiogram images (FA) on September 1, 2021 reveal subtle focal venous vascular leakage of the superior and inferior temporal arcade in the left eye, and in one of the superior arcades in the right eye. Treatment with intravitreal dexamethasone in both eyes showed rapid resolution of leakage in 1 month by November 2021. Three months later (February 2022), there was regression of leakage of the superior temporal arcade in the left eye. **(B)** A heat map displaying the results of a cytokine array that measured the concentration of forty inflammatory cytokines in patient aqueous humor samples collected at multiple points during corticosteroid treatment. Concentrations are plotted on a log2 scale in pg/mL. Grey bars represent concentrations below the threshold of detection. The four MAR samples are shown alongside a non-MAR control cohort (*n* = 10) where the maximum and minimum concentration observed across the cohort are displayed for each cytokine. The detection of the following cytokines in the MAR sample but not in the control cohort suggests a heightened inflammatory state in the MAR patient eye: EGF, GRO, IFNγ, IL-10, IL-11, IL-13, IL-15, IL-17, IL-1β, IL-2, IL-4, IL-5, IL-7, TGFβ1, and TNFα. OD, oculus dexter; OS, oculus sinister.

**Figure 2:**
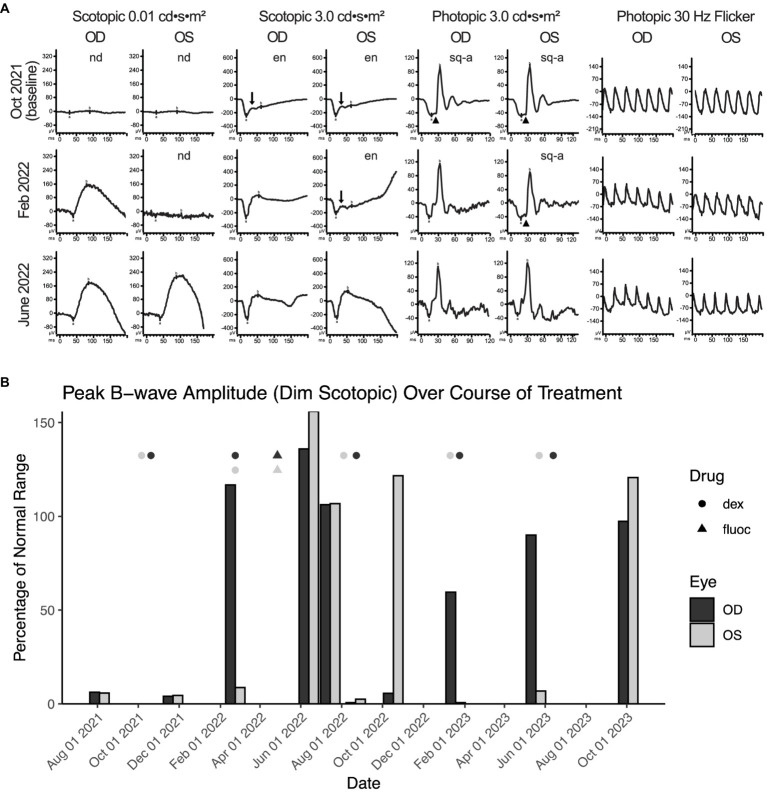
Intraocular corticosteroids restore visual function and alleviate MAR visual symptoms. **(A)** The ffERG waveforms in response to each stimuli of the ISCEV standard are shown at baseline on October 2021, illustrating non-detectable rod-dependent response to the dim scotopic stimuli (0.01 cd•s•m^−2^), electronegative waveform (arrow; b wave amplitude less than a wave amplitude) to the bright scotopic stimuli (3.0 cd•s•m^−2^), squaring of a wave (arrow head; widening of a wave peak) response to the photopic single flash (3.0 cd•s•m^−2^), and normal appearing 30 Hz flicker. Treatment with intravitreal dexamethasone every 16 weeks normalized the right eye by February 2022 and both eyes by June 2022. OD, oculus dexter; OS, oculus sinister; nd, not detectable; en, electronegative; sq-a, squared a-wave. **(B)** Peak b-wave amplitudes from ffERGs collected during corticosteroid implant treatment, shown as a percentage of normal range (lower bound). Eyes were dark adapted for ≥20 min prior to exposure to a 0.01 cd•s•m^−2^ light stimulus. Points mark the dates for intraocular delivery of either dexamethasone (circles) or fluocinolone (triangles) into the left (dark grey) and right (light grey) eyes; stacked points indicate injection to both eyes on the same day. From the first round of dexamethasone delivery, it takes ~3 months to observe the benefit on ffERG b-wave recovery. At baseline, OD and OS achieve only ~6% of the normal range on average. Dex, dexamethasone; fluoc, fluocinolone; OD, oculus dexter; OS, oculus sinister.

The patient presented with BCVA of 20/40 in the right eye and 20/50 in the left eye. Fundus examination was unremarkable, optical coherence tomography showed normal retinal architecture (data not shown), and wide-field autofluorescence was normal at initial presentation and all follow-up encounters (Figure 1A). However, wide-field fluorescence angiography (FA) showed a few areas of subtle focal retinal venous leakage in both eyes (Figure 1A). Wide-field static perimetry revealed severely constricted visual fields in both eyes (Supplementary Figure S1). The ffERG showed non-recordable dim scotopic waveforms, electronegative bright scotopic waveforms, and squared a-waves of the photopic single flash and normal 30 Hz flicker (Figure 2A). Because several studies have reported an improvement of MAR symptoms following intraocular delivery of slow-release corticosteroids (26–28), we administered intraocular slow-release dexamethasone via an intravitreal implant (0.7 mg; AbbVie, North Chicago, IL, United States) to both eyes in October 2021. By February 2022, the patient reported significant improvements to sight, including a reduction in the appearance of “squiggles and stars” in his vision in both eyes, although he felt the left eye had started regressing. Indeed, the ffERG results indicated that the right eye dim scotopic b-wave amplitude had been restored to 116% of the normal range (from 6% at baseline), the electronegative character of the bright scotopic waveform had normalized, and the squared a-wave of the photopic waveform had resolved (Figure 2, for timelines see Supplementary Tables S1, S2). The FA also showed resolution of the leakage in the right eye (Figure 1A). In the left eye, the ffERGs resembled the baseline (Figure 2) and the FA showed recurrence of leakage in the superior arcade, confirming regression of the left eye (Figure 1A). Dexamethasone injections were repeated in both eyes in February of 2022. To potentially reduce the frequency of intraocular dexamethasone injections (every ~3 months), intravitreal fluocinolone acetonide implants (0.18 mg, EyePoint Pharmaceuticals; Watertown, MA, United States), which have a 2–3-year efficacy period, were injected in both eyes in April 2022. Results from the ffERG in June 2022 demonstrated restoration of the dim scotopic b-wave amplitude to 136 and 156% of normal range for the right and left eyes, respectively, and resolution of the bright scotopic electronegative waveform and photopic squared a-wave in the left eye (Figure 2; Supplementary Table S2). Moreover, widefield static perimetry revealed significant expansion and improvement of visual sensitivity in both eyes (Supplementary Figure S1). However, these benefits regressed 2–3 months later. In August 2022, an ffERG revealed that b-wave amplitudes in the right and left eye had fallen to 1 and 2% of the normal range, respectively. Unfortunately, fluocinolone acetonide implant monotherapy was not effective and dexamethasone treatment was reinitiated with repeat injections for both eyes in August of 2022. Two months later in October of 2022, the b-wave in the left eye was restored to 122% of the normal range, while the right eye remained at 6% (Figure 2; Supplementary Tables S1, S2). With continued routine dexamethasone implants every 16 weeks, there was a sustained effect of improved visual symptoms, visual fields, and normalization of the ffERG in both eyes. During the course of treatment, steroid-induced elevated intraocular pressures of 32 and 28 mmHg were observed in the right and left eye, respectively, which normalized with pressure-lowering eye drops (dorzolamide/timolol) twice per day. The patient also developed cataract in both eyes, but with cataract surgery and sustained intravitreal steroids, his BCVA stabilized to 20/30 in both eyes.

### 3.2 Inflammatory cytokine levels are augmented within the aqueous humor

To cause MAR symptoms, TRPM1 autoantibodies must bind and inhibit the TRPM1 ion channel in the membrane of ON-BCs (5, 6). Yet to reach the ON-BCs, the autoantibodies must first breach the blood-retinal barrier (BRB), which, under healthy conditions, restricts the passage of macromolecules from the circulation into the retinal tissue (29, 30). Currently, it is not understood how TRPM1 autoantibodies cross the BRB to reach the retina. Several studies report that inflammation in or near the eye can negatively impact the integrity of the BRB (31–37), which may present an avenue for circulating proteins, such as autoantibodies, to access this restricted space. To assess the inflammatory status of the patient’s eyes at several stages of treatment, the concentrations of forty inflammatory cytokines were examined in aqueous humor samples using a cytokine array (Figure 1B). Fifteen cytokines, which were undetectable in a non-MAR control cohort, appear elevated in the MAR patient, several of which have been implicated in regulating BRB integrity, including IL-1β, IL-17A, TNFα, and VEGF-A (Figure 1B) (32–35, 38). In accordance with the heightened inflammatory state, evidence of focal retinal venous leakage indicative of a compromised BRB was visible in the patient’s fluorescein angiograms on September 1, 2021 (Figure 1A). Over 2 months later in November 2021, the focal retinal venous leakage appeared resolved, following the initiation of dexamethasone treatment on October 6, 2021. By February 23, 2022, leakage reappeared at one site in the left eye within the superior arcade, which correlated with regression of symptoms and abnormal ffERG in the left eye.

### 3.3 TRPM1 autoantibodies persist in patient serum throughout immunotherapy and vision treatment

**Figure 3:**
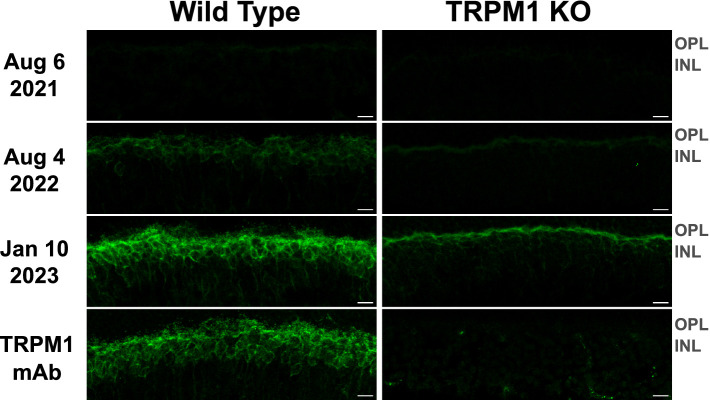
TRPM1 autoantibodies persist in patient serum throughout immunotherapy and vision treatment. Retina sections from WT and TRPM1 knockout mice were incubated with patient serum (diluted 1: 1000) collected on three different dates or with an anti-TRPM1 mouse monoclonal antibody (diluted 1:2000). Anti-retinal autoantibodies in the sera were then visualized by incubating the tissue with an Alexa Fluor 488-conjugated anti-human secondary antibody (green). The anti-TRPM1 monoclonal antibody was visualized using an Alexa Fluor 488-conjugated anti-mouse secondary antibody (green). In all three serum draws, autoantibodies were seen to label bipolar cells. However, the intensity of anti-bipolar cell staining was noticeably higher on sections labeled with the sera from 08/04/22 and 01/10/23. The ON-BC labeling visible in the WT retina is significantly reduced in the TRPM1 KO retina, indicating the majority of ON-BC labeling in the WT retina is caused by autoantibodies against TRPM1. Residual bipolar cell staining in the TRPM1 KO retina is likely caused by additional anti-retinal autoantibodies against other antigens. The similarity in the staining pattern between the anti-TRPM1 monoclonal antibody and the MAR serum on the WT retina is further evidence that the retinal autoantibodies in the MAR patient serum primarily target TRPM1. Scale bars = 10 μm; ONL, outer nuclear layer; OPL, outer plexiform layer; INL, inner nuclear layer.

To detect retinal autoantibodies circulating in the patient’s blood, diluted serum from three separate blood draws was applied to cryosections of mouse retina and bound antibodies were detected with a fluorescent anti-human secondary antibody. Retinal bipolar cells were labeled with all three serum draws, with much brighter labeling produced by the sera from the two later collection dates (Figure 3). This increase in signal strength may be explained by an increase in autoantibody titer, an increase in antibody affinity for retinal autoantigens, or both. When applied to retinal cryosections from TRPM1-KO mice, bipolar cell labeling with the MAR serum was dramatically reduced, indicating that anti-TRPM1 autoantibodies comprise the majority of anti-retinal autoantibodies in the patient’s serum (Figure 3). Further supporting that anti-TRPM1 autoantibodies dominate the composition of retinal autoantibodies, the bipolar cell staining pattern produced by this patient’s serum closely resembles that of an anti-TRPM1 mouse monoclonal antibody (Figure 3). Despite an obvious reduction in immunofluorescence intensity on retina sections from the TRPM1 KO mouse compared to the wild type, anti-retinal labeling is not entirely absent, implicating the presence of autoantibodies against additional retinal antigens in the patient’s serum (Figure 3). Furthermore, the immunofluorescence on the TRPM1-KO retina sections is brighter for the later blood draws indicating an increase in titer and/or affinity of these additional anti-retinal autoantibodies over time. Supplementary Figure S2 shows the ffERG recorded on the same dates as the serum draws.

### 3.4 Patient autoantibodies target a region of TRPM1 encoded by exons 8–10

**Figure 4:**
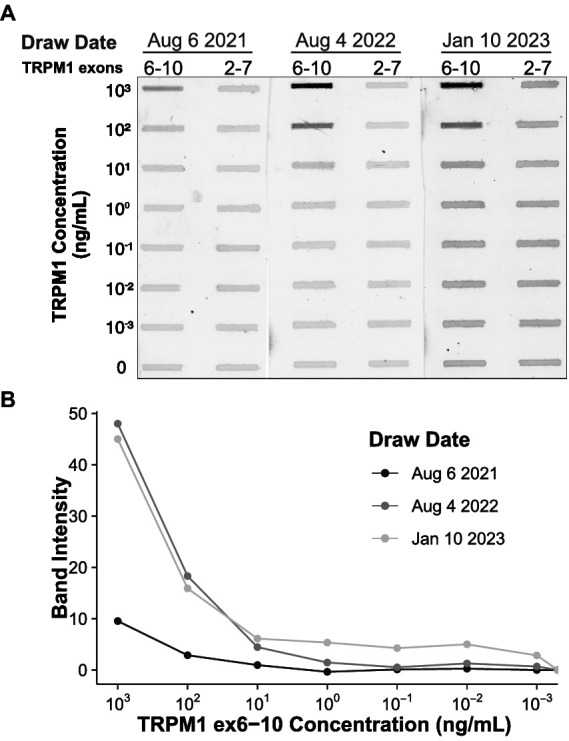
Patient autoantibodies target a region of TRPM1 encoded by exons 8–10. **(A)** Decreasing concentrations of two recombinant TRPM1 polypeptides were applied to a PVDF membrane in triplicate. The two polypeptides, encoded by exons 6–10 and exons 2–7 of TRPM1, respectively, share two exons of sequence overlap. Diluted patient sera from three different collection dates were then applied to the PVDF, and reactivity with the recombinant polypeptides was visualized with an anti-human secondary antibody conjugated to a near-infrared fluorophore. The signal intensities of the slot immunoblot bands are quantified in **(B)**. Autoantibodies in the patient sera exclusively react with the TRPM1 polypeptide encoded by exons 6–10. Sera collected on August 4, 2022 and January 10, 2023 yield more intense staining, either due to higher autoantibody concentration, higher autoantibody affinity, or both.

In characterizing previous MAR patient sera, we mapped the immunoreactive epitope targeted by TRPM1 autoantibodies to a region in the N-terminal, cytoplasmic domain of TRPM1, amino acids 337–380 (encoded by exons 8–10) (Supplementary Figure S3) (39). Using a slot immunoblot assay in which patient serum is reacted with purified TRPM1 polypeptides, we found that autoantibodies in the serum of the current patient also bind to this region of TRPM1. Sera from both the current patient and a previous MAR patient gave nearly identical results on the slot immunoblot, reacting with a TRPM1 polypeptide encoded by exons 6–10, but not with a polypeptide encoded by exons 2–7 (Figure 4A). In agreement with the retinal immunofluorescence results, sera from August 4, 2022 and January 10, 2023 reacted more strongly with the purified TRPM1 polypeptide than the serum from August 6, 2021 (Figure 4B). Despite the comparatively low titer/affinity of anti-TRPM1 autoantibodies in the August 6, 2021 serum, the patient began reporting vision changes as early as April 2021. Consistent with the slot immunoblot results, the patient’s serum labels TRPM1 ex6-10 but not TRPM1 ex2-7 when both polypeptides are heterologously expressed by HEK293T cells (Supplementary Figure S4).

## 4 Discussion

Melanoma-associated retinopathy is a rare paraneoplastic syndrome that affects individuals with cutaneous melanoma, causing visual impairment due to a humoral immune response against ON-BC neurons in the retina. A definitive MAR diagnosis requires assessment of visual dysfunction and autoimmune activity. The bright scotopic ffERG b-wave amplitude provides a useful assessment of ON-BC function and serves as a proxy for the degree of visual impairment. To complement the ffERG, immunofluorescent detection of anti-TRPM1 autoantibodies in the serum indicates the presence and intensity of the immune response against ON-BCs. Together, these tests can be used to monitor disease progression and response to ocular or systemic treatment, such as intraocular corticosteroids or cancer immunotherapy. Here we present the first longitudinal case report of a MAR patient that integrates ffERG recordings, serum autoantibody measurements, aqueous humor cytokine profiling, and the patient’s treatment record. The synthesis and integration of these data shed new light on poorly understood aspects of MAR pathogenesis which may prove useful to the greater community of autoimmune retinopathy researchers and clinicians.

First, although the patient developed symptoms in April 2021 (Supplementary Table S1), we note that ON-BC autoantibodies were barely detectable by immunohistochemistry or immunoblot in the first serum draw from August 6, 2021 (Figures 3, 4A). Consistent with the patient’s symptoms, the ffERG recorded on August 4, 2021 revealed an undetectable dim scotopic waveform and electronegative bright scotopic waveform in both eyes, indicating severe bipolar cell dysfunction (see Supplementary Figure S2). Together, these findings indicate that even low levels of circulating ON-BC autoantibodies can profoundly impact vision. Next, between August 6, 2021 and August 4, 2022, there was a substantial increase in detectable TRPM1 autoantibodies in the patient’s serum (Figures 3, 4), which preceded a melanoma relapse (leptomeningeal disease) in September 2022. This spike in retinal autoantibodies occurred in the absence of cancer immunotherapy, though other reports suggest immunotherapy potentiates autoimmune retinopathy (16, 40, 41). Our patient underwent ~17 months of nivolumab and pembrolizumab therapy before reporting visual symptoms (Supplementary Table S1), whereas other studies report the onset of visual dysfunction after 3–6 weeks of immunotherapy. Therefore, any direct effect of the immunotherapy on visual function appears unlikely in this case. However, in one previous study, a patient who developed MAR visual symptoms after 18 months of pembrolizumab reported greatly improved vision 5 days after stopping treatment. Our patient reported no such improvement to vision after stopping pembrolizumab in August 2021; however, visual function improved after dexamethasone treatment 2 months later. One possible explanation for the autoantibody surge is that the melanoma relapse reinvigorated the immune response against the cancer, leading to increased production of autoantibodies. A progressive increase in the immunofluorescent labeling intensity on the TRPM1 KO retina sections across serum draws demonstrates the development of additional anti-retinal autoantibodies, consistent with a reinvigorated immune response (Figure 3). Increased autoantibody levels in the serum may therefore serve as useful predictors for the progression of cancer prior to PET or CT imaging but are not necessarily linked to cancer immunotherapy.

In agreement with previous case reports on MAR, our patient responded positively to intraocular dexamethasone implants, which restored the normal ffERG and correlated with significant improvement in visual fields (Figure 2; Supplementary Figure S1) (26–28). The abnormal visual fields and the improvement with treatment indicate that visual dysfunction is directly due to the inner retinopathy associated with MAR. While ffERG b-wave amplitudes appeared maximally restored during June through August 2022 (Figure 2), high levels of TRPM1 autoantibodies remained detectable in the patient’s serum during this same period (Figures 3, 4), confirming that circulating TRPM1 autoantibodies alone are not sufficient to cause visual impairment (19). Instead, these data suggest additional pathological factors may be required for autoantibodies to inhibit retinal function. Given that the blood-retinal barrier (BRB) typically prevents antibodies and other macromolecules from accessing the retina, anti-retinal autoantibodies could circulate through the blood yet be restricted from ON-BCs, in which case no visual impairment would occur (19, 37). This concept is supported by a previous study demonstrating the presence of TRPM1 autoantibodies in patients without self-reported visual symptoms (19). Anti-retinal autoantibodies must first circumvent this barrier before reaching the retina, suggesting that BRB damage or malfunction may be a prerequisite for the development of MAR symptoms.

As inflammation is a known modulator of BRB integrity (32, 33, 35, 36, 38), we sought to examine the inflammatory state of the eye to assess the potential for BRB damage in this MAR patient. Our cytokine array detected a heightened inflammatory state in the patient’s aqueous humor on all three collection dates when compared to a non-MAR control cohort (Figure 1B). Furthermore, several cytokines linked to BRB or blood–brain barrier (BBB) damage appeared elevated in this patient and their persistence could potentially have reduced the integrity of the BRB and enabled autoantibody access to the retina (37). Coinciding with the heightened risk of BRB damage caused by inflammation, fluorescein angiograms taken on September 1, 2021 revealed multiple sites of focal retinal venous leakage to indicate the loss of BRB integrity (Supplementary Figure S2). In response to treatment with intraocular dexamethasone, these leakage sites resolved and the patient’s vision improved, supporting the hypothesis that inflammation damages the BRB, allowing for the passage of autoantibodies and the onset of MAR symptoms. The reappearance of focal retinal venous leakage on fluorescein angiograms February 23, 2022 may reflect the wanning efficacy of the previous dexamethasone implants (left: October 6, 2022, right: October 20, 2022) and the delayed effect of the recently injected implants (left: February 23, 2022, right: February 23, 2022).

The success of intravitreal dexamethasone treatment in restoring the patient’s retinal function, visual fields, and visual acuity is consistent with the notion that inflammation regulates BRB integrity. Intraocular dexamethasone reduces inflammation in the eye, which could allow for the restoration of BRB integrity and the exclusion of autoantibodies from the retina, leading to symptom resolution as seen in our patient. Studies that directly evaluate the effects of inflammation on BRB permeability to antibodies are needed to give credence to this hypothesis. Clinicians and researchers should consider collecting these data to compare results in patients with MAR and other autoimmune retinopathies. In doing so, we approach a deeper understanding of the transition from cancer to autoimmunity, the influence of immunotherapy on MAR progression, and the role of the BRB in autoimmune retinopathy.

## Data Availability

The original contributions presented in the study are included in the article/Supplementary material, further inquiries can be directed to the corresponding author.
