# Supplementary material for: Case report: Longitudinal evaluation and treatment of a melanoma-associated retinopathy patient
Source: Front Med (Lausanne). 2024 Sep 10;11:1445180. doi: 10.3389/fmed.2024.1445180 (PMC11420136; doi:10.3389/fmed.2024.1445180)
Supplement: Supplementary file 1 [file Data_Sheet_1.PDF]

Supplementary Material

1 Supplementary Data

Supplementary Material should be uploaded separately on submission. Please include any supplementary data, figures and/or tables.

Supplementary material is not typeset so please ensure that all information is clearly presented, the appropriate caption is included in the file and not in the manuscript, and that the style conforms to the rest of the article.

2 Supplementary Figures and Tables

For more information on Supplementary Material and for details on the different file types accepted, please see here.

Supplementary Table 1 – Patient examination and treatment history

Date	Diagnosis, Treatment, or Exam	Clinical Notes
February 22, 2019	Detection of metastatic melanoma	FNA + metastatic melanoma. PET shows left groin mass, 2 enlarged L inguinal nodes, 0.7cm LUL nodule
April 2019	Dabrafenib and Trametinib	Neoadjuvant therapy; BRAF V600E inhibitors
July 2019	Resection to NED	Primary found on lower back. WLE of primary and resection of metastatic lymph nodes
October 16, 2019	Brain MRI; PET scan	Brain MRI shows 2 cerebellar legions. PET shows 2 new left pulmonary nodes and right inguinal LN
October 11, 2019	SRS and Nivolumab (PD-1 inhibitor)	Stopped Dabrafenib and Trametinib targeted therapies and transitioned to Nivolumab with stereotactic radiosurgery (SRS).
December 2019	Skin biopsy	Left groin biopsy showed metastatic melanoma
January 14, 2020	Pembrolizumab + PV-10 injections	PV-10 = 10% Rose Bengal, selectively destroys tumor cells. Pembrolizumab = PD-1 inhibitor
July 2, 2020	Pembrolizumab + PV-10 injection	Regressed cutaneous metastases, stopped PV-10, continued pembrolizumab
April 2021	First report of visual symptoms	Symptoms include photopsia, nyctalopia, blue vision, and squiggly lines in the visual field.
May 13, 2021	Immunotherapy on hold	Concerns of optical neuritis or possible vision change as result of immunotherapy
June 8, 2021	Prednisone 1 mg/kg	Treatment for optic neuritis. Completed taper 2 weeks later on 6/22/21. Reported no impact on vision
June 24, 2021	Resumed Pembrolizumab	
August 4, 2021	MAR diagnosis ffERG	Baseline ffERG with electronegative dim scotopic b-wave; suspected MAR.
August 5, 2021	Discontinued Pembrolizumab	Persistently worsening vision, concerns that immunotherapy is potentiating MAR
August 6, 2021	Serum draw	Retinal autoantibodies are extremely faint but detectable.
October 2021	Syncopal Event	Thought to be due to myocarditis. Transitioned to surveillance. Followed by extensive cardiac workup: Ziopatch, CTA coronay, cardiac MRI reveal non-obstructive CAD but no evidence of myocarditis. Cleared to resume immunotherapy.
October 6, 2021	Ozurdex (Dexamethasone), OS	Lasts ~3 months
October 20, 2021	Ozurdex (Dexamethasone), OD	Lasts ~3 months
November 22, 2021	ffERG	

February 23, 2022	Ozurdex (Dexamethasone), OU<br>ffERG	Lasts 2-3 months. Compared to the 11/21 Epsion DTL baseline, OD demonstrates notable improvements in amplitudes of rod-driven response and no longer demonstrates electronegative pattern. OS rod driven responses remain unchanged/similar to prior, including non-detectable responses to dim flash and an electronegative pattern to bright flash. Photopic con-driven responses were stable versus mild fluctuations OU compared to 11/21.
March 1, 2022	Checkup	ffERG on 2/23/22 reveals significant improvement OD. Patient reports significant improvement to vision in both eyes after about 3 months post-Ozurdex, with a reduction in “squiggles and stars” in the left eye and restoration of night vision
April 27, 2022	Yutiq (Fluocinolone acetonide), OU	Lasts 3 years
June 13, 2022	ffERG	Compared to 02/23/22, OD stable and OS has improved significantly.
July 15, 2022	ffERG	Compared to 06/21/22, there was diffuse mild worsening of responses OU, but still better than 02/23/22.
August 4, 2022	Ozurdex (Dexamethasone), OS<br>Serum draw	Indication: states his OS is “going back the way it was.” Retinal autoantibodies are easily detectable.
August 22, 2022	Detection of steroid-induced cataracts OU. Begins Cosopt BID OU<br>ffERG	Cosopt = carbonic anhydrase inhibitor with a beta-adrenergic receptor blocking agent intended to reduce intraocular pressure. BID = “twice daily”. Compared with 07/15/22, rod responses were flat with reversion to electronegative scotopic waveforms and squaring of photopic a waves OU.
August 23, 2022	Ozurdex (Dexamethasone), OD	Indication: return of symptoms
September 1, 2022	MRI brain and spine	Enlargement of inferior right cerebellar mass (3mm to 10mm). Small adjacent mass 4mm. Previously demonstrated enhancing masses in the superior cerebellum not seen. Leptomeningeal disease in lumbar spine and thoracic spine. Lumbar mass = 4cm, thoracic mass = 10mm, causing severe narrowing of spinal canal
September 4, 2022	T6-T7 thoracic laminectomy with resection of intradural extramedullary mass	confirmed metastatic melanoma
October 3, 2022	Complete radiation to T4-T7, L2-L5, and right sided cerebral metastases	
October 7, 2022	CT PET	FDG avid left iliac nodal involvement and osseous involvement of the left scapula and left interior first rib. Low level FDG uptake within left level 1B cervical lymph node, likely reactive. FDG = radiotracer

October 10, 2022	Ipilimumab 3 mg/kg + nivolumab 1 mg/kg	administered every 21 days x 4 cycles
October 17, 2022	ffERG	OS responses have normalized but OD remains abnormal.
December 19, 2022	MRI thoracic and lumbar spine	Nodular enhancement dorsal to L4, less prominent than in 09/01/22. L2-L3 focal moderately severe spinal canal stenosis
December 26, 2022	Brain MRI	2 small cerebral metastases that appear unchanged since 09/01/22.
December 30, 2022	CT PET	Multiple pleural and peripheral nodules and masses that are hypermetabolic
January 10, 2023	Ozurdex (Dexamethasone), OS<br>Serum draw	Lasts ~3 months<br>Retinal autoantibodies are prominent
January 18, 2023	ffERG	Improved responses OD, but worsening OS.
January 24, 2023	Ozurdex (Dexamethasone), OD	Lasts 2-3 months
February 27, 2023	Chest CT	Improvement in pulmonary consolidation
April 17, 2023	MRI Brain; Thoracic/lumbar spine MRI	Increasing size of 2 enhancing right cerebellar metastases. No new lesions in brain or thoracic/lumbar
April, 2023	Cataract extraction and intraocular lens implant	First impacted eye
May 18, 2023	ffERG	Improved responses OD, but no improvements OS.
May 22, 2023	Nivolumab	480 mg every 28 days
May 23, 2023	Ozurdex (Dexamethasone), OS	Lasts ~3 months
May 24, 2023	Cataract extraction and intraocular lens implant	Second impacted eye
June 13, 2023	Ozurdex, OD	Lasts ~3 months
October 3, 2023	ffERG	Huge improvement to OS. OD improved as well

Supplementary Table 2 – Plotted peak electroretinogram b-wave amplitudes and percentage of normal range values for Figure 2.

Date	Eye	Amplitude (μV)	Percent Normal
August 4, 2021	R	10.098	6.27204969
August 4, 2021	L	9.18	5.70186335
November 22 2021	R	6.426	3.99130435
November 22 2021	L	7.344	4.56149068
February 23, 2022	R	188	116.770186
February 23, 2022	L	14	8.69565217
June 13, 2022	R	219	136.024845
June 13, 2022	L	251	155.900621
July 15, 2022	R	171	106.21118
July 15, 2022	L	172	106.832298
August 22, 2022	R	1	0.62111801
August 22, 2022	L	4	2.48447205
October 17, 2022	R	9	5.59006211
October 17, 2022	L	196	121.73913
January 18, 2023	R	96	59.6273292
January 18, 2023	L	1	0.62111801
May 18, 2023	R	145	90.0621118
May 18, 2023	L	11	6.83229814
October 3, 2023	R	156.7	97.3291925
October 3, 2023	L	194.5	120.807453

Supplementary Table 3 – Amino acid sequences of the two human TRPM1 recombinant proteins cloned from M14 cells (Figure 3) and the previously mapped MAR epitope. All references to amino acid and exon positions are given relative to NCBI reference sequence NM_001252020.2

Sequence Name	Amino Acid Sequence
TRPM1ex2-7	MGSSHHHHHHSSGLVPRGSHMARNMKDSNRCCCGQFTNQHIPPLPSATPSKNEEESKQVETQPEKWSVAKHTQSYPTDSYGVLEFQGGGYSNKAMYIRVSYDTKPDSLLHLMVKDWQLELPKLLISVHGGLQNFEMQPKLKQVFGKGLIKAAMTTGAWIFTGGVSTGVISHVGDALKDHSSKSRGRVCAIGIAPWGIVENKEDLVGKDVTRVYQTMSNPLSKLSVLNNSHTHFILADNGTLGKYGAEVKLRRLLEKHISLQKINTRLGQGVPLVGLVVEGGPNVVSIVLEYLQEEPPIPVVICDGSGRASDILSFAHKYCEEGG
TRPM1ex6-10	MGSSHHHHHHSSGLVPRGSHMARNVTRVYQTMSNPLSKLSVLNNSHTHFILADNGTLGKYGAEVKLRRLLEKHISLQKINTRLGQGVPLVGLVVEGGPNVVSIVLEYLQEEPPIPVVICDGSGRASDILSFAHKYCEEGGIINESLREQLLVTIQKTFNYNKAQSHQLFAIIMECMKKKELVTVFRMGSEGQQDIEMAILTALLKGTNVSAPDQLSLALAWNRVDIARSQIFVFGPH
MAR epitope	EGGIINESLREQLLVTIQKTFNYNKAQSHQLFAIIMECMKKKEL

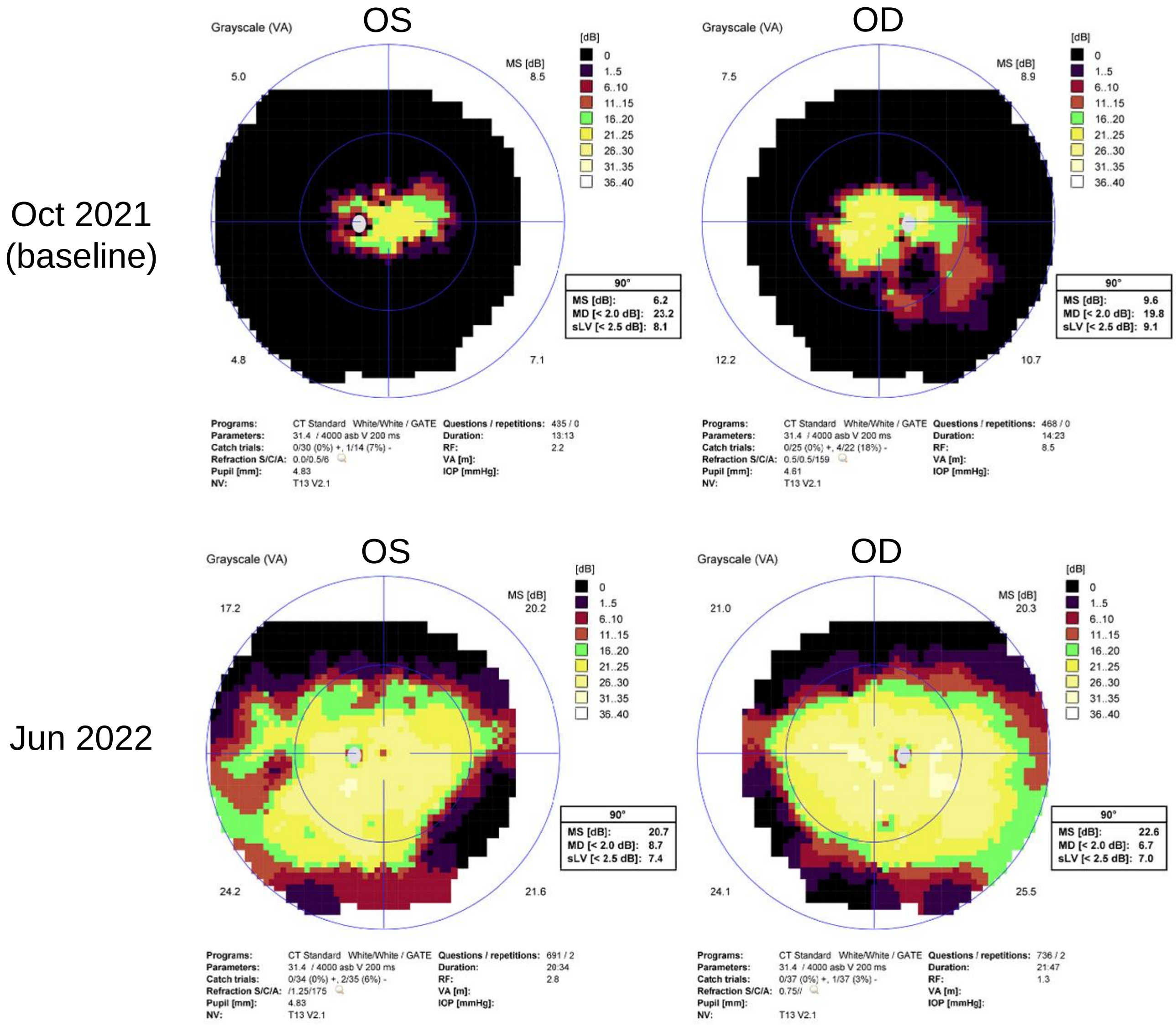

Supplementary Figure 1 – Effect of intravitreal corticosteroids on static perimetry. The full field static perimetry results are shown at baseline in October of 2021, illustrating severely constricted field of sensitivity down to a central field of <45 degrees diameter in both eyes. Treatment with intravitreal dexamethasone every 16 weeks significantly improves the sensitivity of the central field and expands the fields to >90 degrees diameter in both eyes by June of 2022. OD, oculus dexter; OS, oculus sinister.

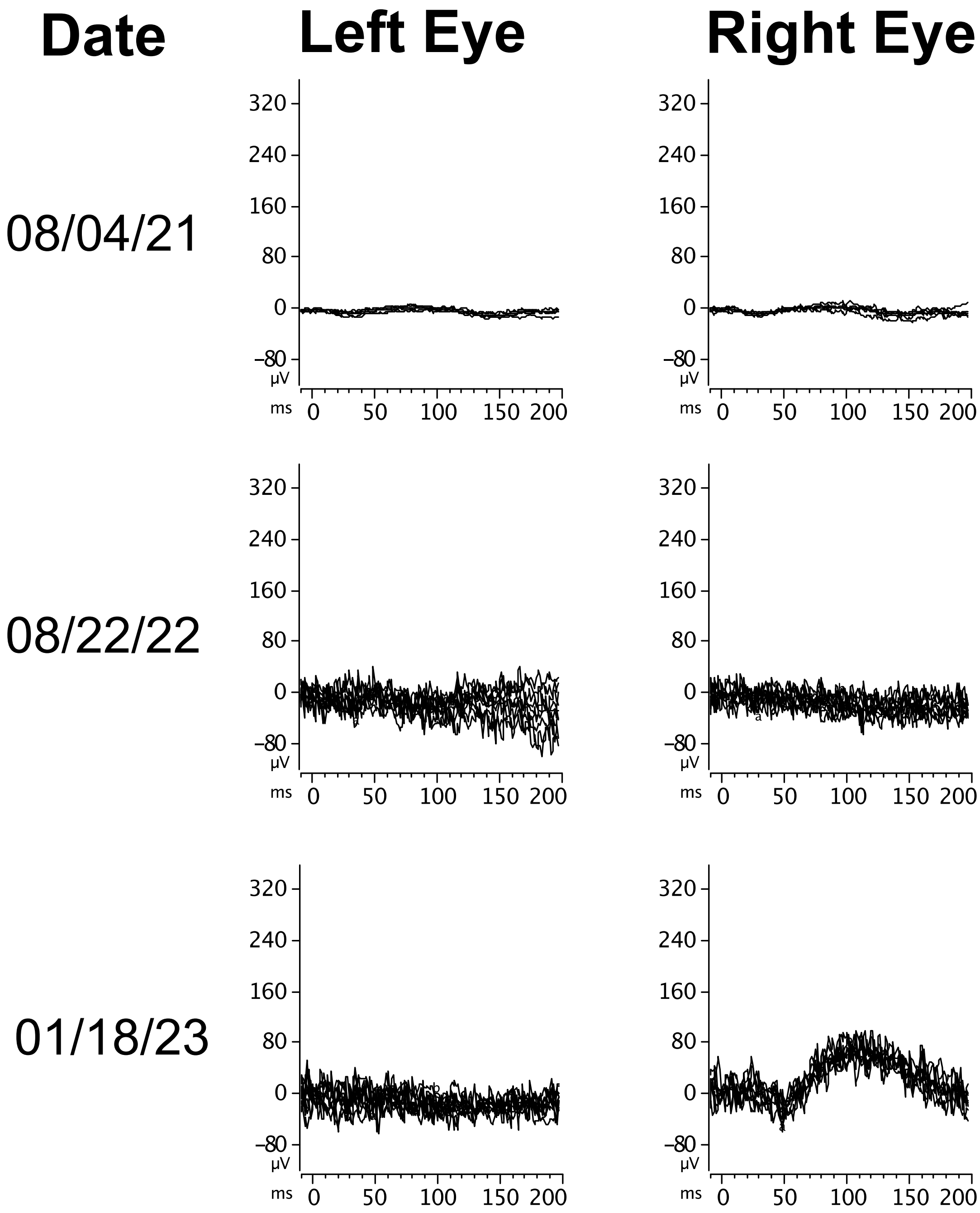

Supplementary Figure 2 – Dark adapted ffERG recordings taken on the same dates as the serum draws.

Examples of raw traces from dark adapted ffERG recordings in the left and right eye used to calculate the peak b-wave amplitudes shown in Figure 1. The recordings shown here correspond to the dates of the serum draws shown in Figures 2 and 3. Multiple recordings are overlayed in each panel.

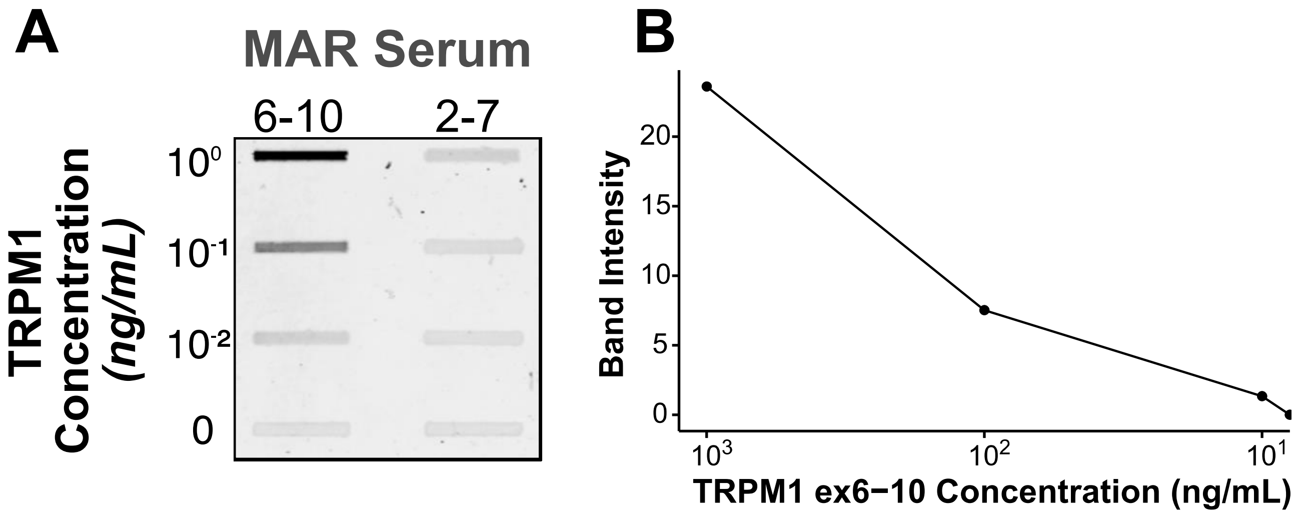

Supplementary Figure 3 – Serum autoantibodies from the current patient target the same TRPM1 epitope as those from a previous MAR patient.

A slot immunoblot in which serial dilutions of two recombinant TRPM1 polypeptides were applied to a PVDF membrane and reacted with diluted serum from a previous MAR patient. The two polypeptides, encoded by exons 6-10 and exons 2-7 of TRPM1, respectively, share two exons of sequence overlap. Serum autoantibody reactivity with the recombinant polypeptides was visualized with an anti-human secondary antibody conjugated to a near-infrared fluorophore. The signal intensities of the slot immunoblot bands are quantified in **B**.

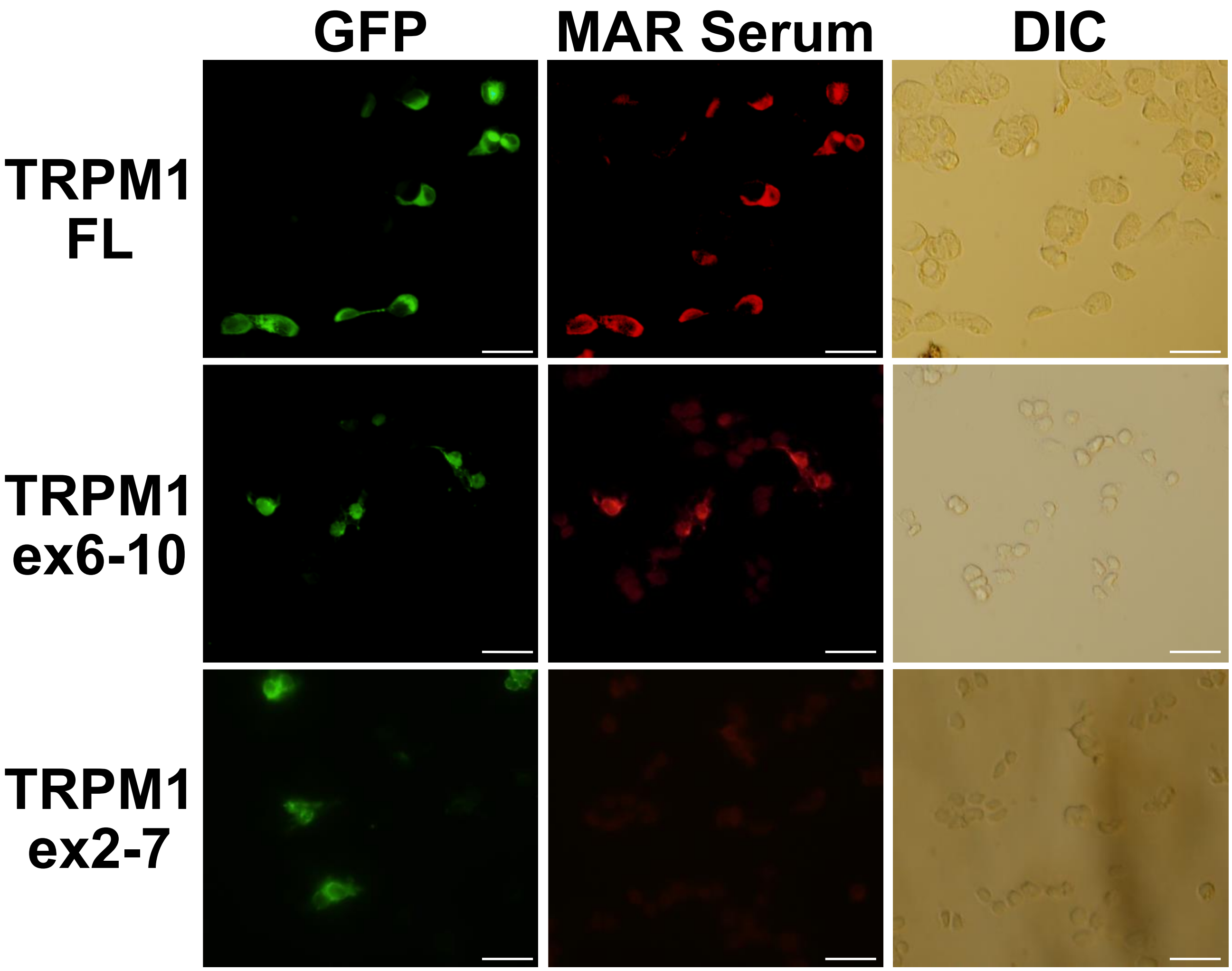

Supplementary Figure 4 – Serum autoantibodies bind recombinant TRPM1 expressed in HEK293T cells.

HEK293 T cells were transfected with one of three GFP-tagged human TRPM1 isoforms encoding the full-length channel (TRPM1 FL), the region encoded by exons 6-10 (TRPM1 ex6-10), or the region encoded by exons 2-7 (TRPM1 ex2-7). Cells were fixed, then incubated with MAR patient serum and TRPM1-reactive autoantibodies were identified using an Alexa Fluor 594-conjugated anti-human secondary antibody. Patient autoantibodies react with the full length TRPM1 and ex6-10 isoforms, but not the ex2-7 isoform, consistent with the slot immunoblot and retina IHC results.

3 Methods

3.1 MAR Sample Tissue Collection

Aqueous humor samples were taken from the front of the patient's eye and were promptly frozen at -80° until use. Whole blood samples were collected in tubes coated with a clotting factor and allowed to clot overnight at 4 ºC. The following day, serum was collected from the surface of the clot and centrifuged at 1,500 x g for 10 minutes. The supernatant was then transferred to a new tube and aliquots kept at 4 ºC for short term use and at -80 ºC for long term storage.

3.2 Slot Immunoblotting

Sequences from the TRPM1 N-terminal cytoplasmic domain were cloned from M14 melanoma cells, expressed in *E. coli*, and purified to produce two polypeptides with partially overlapping sequences (**Supplementary Table 1**). All references to amino acid and exon positions are given relative to NCBI reference sequence NM_001252020.2. The polypeptides were diluted into phosphate buffered saline (PBS) with 0.5% horse serum and 0.05% NaN_3, then applied to a polyvinylidene difluoride (PVDF) membrane using a Bio-Rad Bio-Dot apparatus. The membrane was blocked in Tris-buffered saline with 0.2% Tween-20 (TBST) and 0.5% horse serum for 1 hr at room temperature, then probed with human MAR serum diluted 1:2000 in the same blocking buffer for 1 hr at room temperature. After three, five-minute washes in TBST, the membrane was incubated with an anti-human IRDye 800CW secondary antibody (1:10K). The membrane was washed thrice more, and results were visualized on a Licor Odyssey infrared imaging system (LI-COR; Lincoln, NE, USA).

3.3 Tissue Preparation for Immunofluorescence

The cornea and lens were removed from freshly dissected mouse eyes and the remaining eyecups (retina and sclera) were immediately fixed by immersion in ice-cold 4% paraformaldehyde for 30 min. Following fixation, the eyecups were washed in ice-cold PBS, then cryoprotected by successive incubation in 10%, 20%, and 30% sucrose for 1 hr each at 4 ºC. Cryoprotected eyecups were embedded in optimum cutting temperature (OCT) medium (Sakura Finetek; Tokyo, Japan) and stored at -80 ºC until sectioning, at which point 20 µm transverse sections were cut using a cryostat. Retinal sections were mounted on Superfrost slides (Thermo Fisher Scientific; Waltham MA, USA), air dried, and stored at -20 ºC or -80 ºC until use.

3.4 Retina Section Immunostaining

Frozen cryosections were thawed, then incubated in antibody incubation solution (AIS; 3% horse serum, 0.5% Triton X-100, 0.025% NaN3 in PBS) for 30 min at room temperature to hydrate, permeabilize, and block the tissue. Sections were then incubated with MAR serum or a mouse TRPM1 antibody (Agosto et al., 2014) diluted in AIS for 1 hr at room temperature. After 3 washes in room temperature PBS, the sections were incubated for 1 hr with secondary antibodies conjugated to Alexa Fluor 488 (1:1000) and washed three more times. Finally, mounting medium was applied to the samples, followed by coverslips, and the results were visualized by confocal microscopy.

3.5 Confocal Imaging

Confocal micrographs were taken with a Leica TCS SP8 X confocal microscope (Leica; Wetzlar, Germany) using Leica HC PL APO CS2 40X/1.3 and HC PL APO CS2 63X/1.40 oil immersion

objectives (Leica; Cat# 506358, Cat# 15506350). FIJI was used to adjust brightness and contrast and generate Z-projections from image stacks.

3.6 Cell Culture, Transfection, and Immunocytochemistry

HEK293T cells (RRID: CVCL_0063) cultured in DMEM with 10% fetal bovine serum were seeded onto polylysine-coated coverslips contained in 24-well tissue culture plates. Using Effectene (Qiagen, Valencia, CA, USA), cells were transfected with pEGFP-C3 plasmids encoding TRPM1 isoforms with EGFP fused to their N-termini. The following day, the cells were fixed for 5 minutes in 4% paraformaldehyde at room temperature, then permeabilized and blocked with AIS for 30 minutes at room temperature. The cells were then incubated with diluted patient serum for 1 hour at room temperature, washed 3 times in PBS, and incubated with an Alexa Fluor 594-conjugated anti-human secondary antibody for 1 hour at room temperature. After 3 additional PBS washes, the coverslips were mounted onto Superfrost slides and imaged.

3.7 Visual Diagnostics

At regular intervals, the patient underwent ophthalmic examination and testing, which included best-corrected visual acuity, spectral-domain optical coherence tomography (Heidelberg, Franklin, MA, USA), wide-field fundus photography, wide-field fundus autofluorescence (AF), wide-field fluorescein angiography (FA; Optos, Marlborough, MA, USA), wide-field static perimetry (Octopus 900, Haag-Streit, Switzerland), and full-field electroretinography (Diagnosys LLC, Lowell, MA, USA) in accordance with the International Society for Clinical Electrophysiology of Vision (McCulloch et al., 2015). Data were collected with either Burien Allen (BA) or Espion DTL electrodes; data collected on BA electrodes were multiplied by the median ratio of the peak b-wave amplitudes (BA/DTL) measured in a control cohort (n=9) to transform them for comparison with data collected on DTL electrodes. From dark adapted ffERGs with a 0.01 $cd \cdot s \cdot m^{-2}$ stimulus intensity, peak b-wave amplitudes were divided by the 2.5^{th} percentile (161 µV) of the empirically determined normal range to yield “percentage of normal range.”

3.8 Cytokine Array Preparation and Quantification

MAR serum and aqueous humor samples were diluted 2X in incubation buffer, then applied to a commercial slide array (Ray Biotech; Cat# QAH-DED-1-1; Corners, GA, USA). The array was prepared and incubated with MAR samples and cytokine standards according to the manufacturer’s protocol. Upon completion of the protocol, the array was shipped to Ray Biotech for quantification. Raw data was processed and analyzed using Ray Biotech’s QAH-DED-1 Array Analysis Tool.
